# Plasticity of cone photoreceptors in adult zebrafish revealed by thyroid hormone exposure

**DOI:** 10.1038/s41598-023-42686-x

**Published:** 2023-09-21

**Authors:** Ashley A. Farre, Preston Thomas, Johnson Huang, Rachel A. Poulsen, Emmanuel Owusu Poku, Deborah L. Stenkamp

**Affiliations:** 1 Department of Biological Sciences, University of Idaho, Moscow, ID 83844-3015 USA; 2 WWAMI Medical Education Program, University of Washington School of Medicine, University of Idaho, Moscow, ID USA; 3 University of Washington School of Medicine, Spokane, WA USA; 4 Brigham Young University, Idaho, Rexburg, ID USA

**Keywords:** Developmental biology, Neuroscience

## Abstract

Vertebrate color vision is predominantly mediated by the presence of multiple cone photoreceptor subtypes that are each maximally sensitive to different wavelengths of light. Thyroid hormone (TH) has been shown to be essential in the spatiotemporal patterning of cone subtypes in many species, including cone subtypes that express opsins that are encoded by tandemly replicated genes. TH has been shown to differentially regulate the tandemly replicated *lws* opsin genes in zebrafish, and exogenous treatments alter the expression levels of these genes in larvae and juveniles. In this study, we sought to determine whether gene expression in cone photoreceptors remains plastic to TH treatment in adults. We used a transgenic *lws* reporter line, multiplexed fluorescence hybridization chain reaction in situ hybridization, and qPCR to examine the extent to which cone gene expression can be altered by TH in adults. Our studies revealed that opsin gene expression, and the expression of other photoreceptor genes, remains plastic to TH treatment in adult zebrafish. In addition to retinal plasticity, exogenous TH treatment alters skin pigmentation patterns in adult zebrafish after 5 days. Taken together, our results show a remarkable level of TH-sensitive plasticity in the adult zebrafish.

## Introduction

Photoreceptors are a class of light-sensing neurons in the vertebrate retina. While rod photoreceptors are predominantly responsible for low-light, low-acuity vision, cone photoreceptors mediate high-acuity color vision. In many vertebrates, separate subpopulations of cones express distinct cone opsins: proteins that, together with a chromophore, form visual pigments that are maximally sensitive to specific wavelengths of light. The presence of multiple cone subtypes, each expressing a unique opsin and therefore sensitive to particular wavelengths of light, serves as the basis of color vision^1, 2^, although in some cases rods may also participate^3, 4^.

Humans possess three cone subtypes (red-, green-, and blue-sensing) which express long wavelength sensitive (LWS), middle wavelength sensitive (MWS), and short wavelength sensitive (SWS) opsins, respectively^5^. The genes encoding the human LWS and MWS opsins are arranged in tandem on the X chromosome^6^, and the mechanism by which they are regulated remains largely unknown. Several models for the regulation of the human *LWS* and *MWS* opsin genes have been suggested^7–9^; however, the study of tandemly replicated opsin genes is challenging due to the high sequence similarity of the primate LWS and MWS opsin proteins, mRNAs, and genes^10^ and because the only non-human mammals known to express tandemly replicated opsins are other primates and bats^11^. The genomes of teleost fish, however, harbor numerous instances of tandemly replicated cone opsin genes^4,11–13^. For example, zebrafish possess two sets of tandemly replicated opsins, the long wavelength sensing *lws* opsin genes (*lws1* and *lws2*) and the middle wavelength sensing *rh2* opsin genes (*rh2-1, rh2-2, rh2-3, rh2-4*)^13^. The zebrafish *lws* opsin genes and the human *LWS/MWS* opsin genes evolved from a common ancestral *LWS* opsin gene^11^. As such, the zebrafish serves as a suitable vertebrate model organism for the study of tandemly replicated opsin gene regulation.

Previous work using the zebrafish, other model organisms, and retinal organoids derived from human embryonic stem cells (ESCs) or induced pluripotent stem cells (iPSCs) has shown that thyroid hormone (TH) is essential in determining cone subtype identity and patterning^14–20^. Recent studies from our lab demonstrated that TH regulates the expression of tandemly replicated opsin genes in the zebrafish. For both the *lws* and *rh2* arrays, TH was shown to promote the expression of the long wavelength-shifted member(s) of the array at the expense of the more short wavelength-shifted member(s), and the athyroid condition resulted in increased expression of the more short wavelength-shifted *lws2* at the expense of *lws1* in larvae and juveniles^14^. These results added to a wide array of evidence showing TH has a conserved role in long wavelength-shifting retinal gene expression^21–25^. Further, treatment of zebrafish larvae with exogenous TH was shown to induce identified LWS2 cones to begin expressing an *lws1* reporter^14^. This phenomenon, which we refer to as “opsin switching” indicates that gene expression in individual larval LWS cones is plastic to TH treatment. Indeed, the expression of *lws1* and *lws2* in juveniles can also be altered by exogenous TH treatment, showing this plasticity remains at least through the juvenile stage^14^.

The two LWS cone subtypes in the zebrafish, LWS1 and LWS2, are known to differ transcriptionally beyond opsin expression^20,26^. We have shown that some of these differentially expressed transcripts are also plastic to TH treatment. For example, *gngt2a* and *gngt2b* are paralogous genes encoding gamma subunits of transducin (a heterotrimeric g-protein component of the phototransduction signaling cascade) and are enriched in LWS1 and LWS2 cones, respectively. *Gngt2b* is downregulated by TH treatment in larval zebrafish, as is *lws2*, while the expression domain of *gngt2a* expands in response to TH treatment, although transcript abundance does not change^20^. These results indicate that transcriptional heterogeneity between LWS cones as well as opsin expression itself is likely mediated in part by TH.

TH serves as an endocrine signal. The active form of TH is triiodothyronine (T3). Thyroxine (T4) is a less active form of TH and can be converted to T3 within cells of target tissues by the membrane-bound enzyme deiodinase 2 (Dio2). The thyroid gland primarily synthesizes T4, which is bound by carrier proteins and transported to tissues, where it enters cells through thyroid hormone transporters such as MCT8^27–29^. T3 can bind to nuclear hormone receptors called TH receptors (TRs) to regulate gene expression. TRs can form homodimers or heterodimers with retinoid X receptors (RXRs), which bind retinoic acid^30,31^.

In teleost fish, including zebrafish, TH signaling is known to mediate changes in morphology, skin pigmentation, feeding strategies, and expression of cone opsin genes, among other features, as fish progress through life history changes. The zebrafish larval-to-juvenile transition, following the time of yolk resorption, is characterized by changes in skull morphology that facilitate jaw protrusion for feeding^32^, the formation of the striped pattern of skin pigmentation^33^, and expansion of the *lws1* expression domain in the retina at the expense of the *lws2* domain^14,34^. The juvenile stage in zebrafish is considered to begin at approximately 30 days post-fertilization^35^). The juvenile-to-adult transition corresponds to the time of sexual maturity (~ 3 months)^35^ and includes further changes in skin pigmentation^33^ and opsin expression^34^.

In this study, we aimed to determine the reach of TH-mediated transcriptional plasticity by investigating whether the cones of adult (0.5–1.5-year-old, reproductively mature) zebrafish remain plastic to TH treatment. Cone subtype patterning in larval and juvenile fish is dynamic and likely regulated by both TH and retinoic acid (RA) signaling^14,36^. However, the pattern of cone subtypes in adult fish, or after natural changes in opsin expression have taken place, is thought to be stable^34,37,38^. T3 is present at detectable levels in the eyes of adult zebrafish, as is the T4 to T3-converting enzyme Dio2, indicating the possibility that TH may be involved in the homeostatic maintenance of cone subtype patterning in the adult zebrafish^39^. Other studies have shown that continued TH signaling is important to maintain skin pigment patterning in adult zebrafish^33^. It is unknown, however, whether exogenous TH treatment can change established cone subtype patterns in the adult zebrafish retina.

Our results in the current study indicate that cone subtype patterning in the retina is indeed plastic to exogenous TH treatment even in the adult zebrafish, and this plasticity occurs in as little as 7 h. We also found that the kinetics of the TH-induced changes in gene expression varied between transcripts. Specifically, the expression domain and transcript abundance of *lws1* changed more rapidly than those of other genes, including *lws2*. Additionally, we found that skin pigmentation patterns in adult zebrafish are also plastic to exogenous TH treatment. Taken together, our results show a remarkable level of TH-mediated plasticity in the adult zebrafish and underscore the importance of TH in maintaining homeostasis in cell patterning.

## Results

### T4 treatment alters topography of lws1 and lws2 reporter patterning in adult lws:PAC(H) transgenic fish

The *lws:PAC(H)* transgenic reports *lws1* expression with GFP and *lws2* expression with dsRedExpress (“RFP”). This line [*Tg(LWS1/GFP-LWS2/RFP-PAC(H))#430,* (kj15Tg)] has been shown to recapitulate the characteristic pattern of *lws1* and *lws2* mRNA-expressing cones in the adult zebrafish, in which *lws1* is expressed in the ventral and nasal periphery, with some expression in the dorsal periphery, and *lws2* is expressed centrally and dorsally^34,40^. Further, the *lws1* and *lws2* reporters reproduce the response of the native transcripts to TH^14^. In larval zebrafish, native *lws1* mRNA and the GFP reporter of *lws1* in *lws:PAC(H)* show increases in size of their expression domains in response to 100 nM TH treatment while native *lws2* mRNA and the RFP reporter of *lws2* in *lws:PAC(H)* domains decrease^14^. Previous studies have shown that treatment of athyroid juvenile *lws:PAC(H)* zebrafish with T4 rescues GFP (*lws1*) expression, indicating that the reporter construct remains plastic to TH treatment at the juvenile stage, similar to the behavior of the native *lws* array^14^. As such, we reasoned it would be appropriate to investigate plasticity using the *lws:PAC(H)* reporter line.

**Figure 1:**
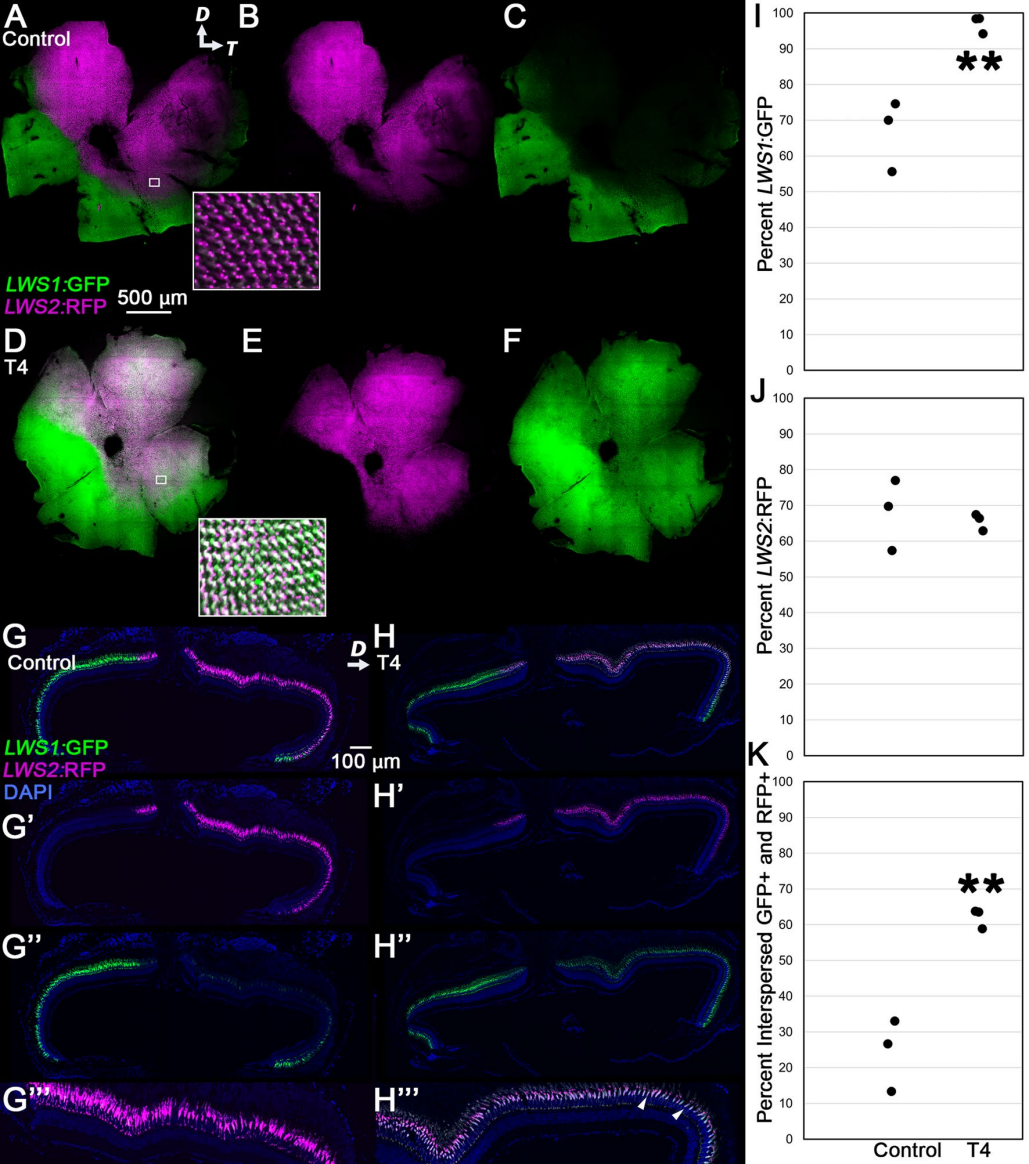
Topography of *lws1* and *lws2* reporter patterning in adult *lws:PAC(H)* transgenic fish, in which GFP reports *lws1* expression and RFP reports *lws2* expression. (**A**–**F**) Projections of representative whole, imaged retinas from fish treated with 0.01% NaOH (control, **A**–**C**) or 386 nM T4 in 0.1% NaOH (treated, **D**–**F**) for 5 days. Insets show indicated regions enlarged by 1400%. (**A**,**D**) All imaging channels merged. (**B**,**E**) *lws2*:RFP. (**C**,**F**) *lws1*:GFP. Note expanded GFP expression domain in response to T4 (**D**–**F**). (**G**,**H**) Projections of representative sectioned retinas from fish treated with NaOH (control, **G**–**G″′**) or T4 (treated, **H**–**H″′**). (**G**,**H**) All imaging channels merged. (**G′**,**H′**) *lws2*:RFP. (**G″**,**H″**) *lws1*:GFP. (**G″′**,**H″′**) enlarged image of *lws2*:RFP-containing region. Arrows indicate cells coexpressing GFP and RFP (see also Supplementary Fig. S1). (**I**–**K**) Analysis of areas of expression domains, n = 3 for each group. (**I**) Percent of retina occupied by GFP+ cells (*p* = 0.0068, t-test). (**J**) Percent of retina occupied by RFP+ cells (*p* = 0.6910, t-test). (**K**) Percent of retina occupied by GFP+ and RFP+ cells interspersed or coexpressing (*p* = 0.0033, t-test). Fisher’s Exact Test was used to test for overall differences: *p* = 0.0034 with a 3 × 2 contingency table. *D* dorsal, *T* temporal.

Confocal imaging and subsequent expression domain area analysis showed that after 5 days of treatment with T4, the GFP (*lws1*) expression domain significantly expanded in comparison to controls (Fig. 1A–F). In control retinas, the GFP domain was found to include approximately 60% of the retina, while in T4 treated retinas, the GFP domain covered over 90% of the retina (Fig. 1I). In contrast, the RFP (*lws2*) domain remained similar in both groups (approximately 60%, Fig. 1J). This resulted in an increased region of interspersed and/or coexpressing GFP + and RFP + cones (Fig. 1K). Five-micron tissue sections (Fig. 1G–G″′,H–H″′) showed that in retinas from T4-treated fish, the majority of RFP expressing cells also expressed GFP (Fig. 1H″′; Supplementary Fig. S1). This coexpression could indicate that both members of the transgenic array were being transcribed or that only GFP was transcribed, but RFP (protein) had not yet been degraded. In order to distinguish between these possibilities, our next studies focused on monitoring endogenous mRNA.

### Five days of T4 treatment induce widespread cone transcriptional changes

**Figure 2:**
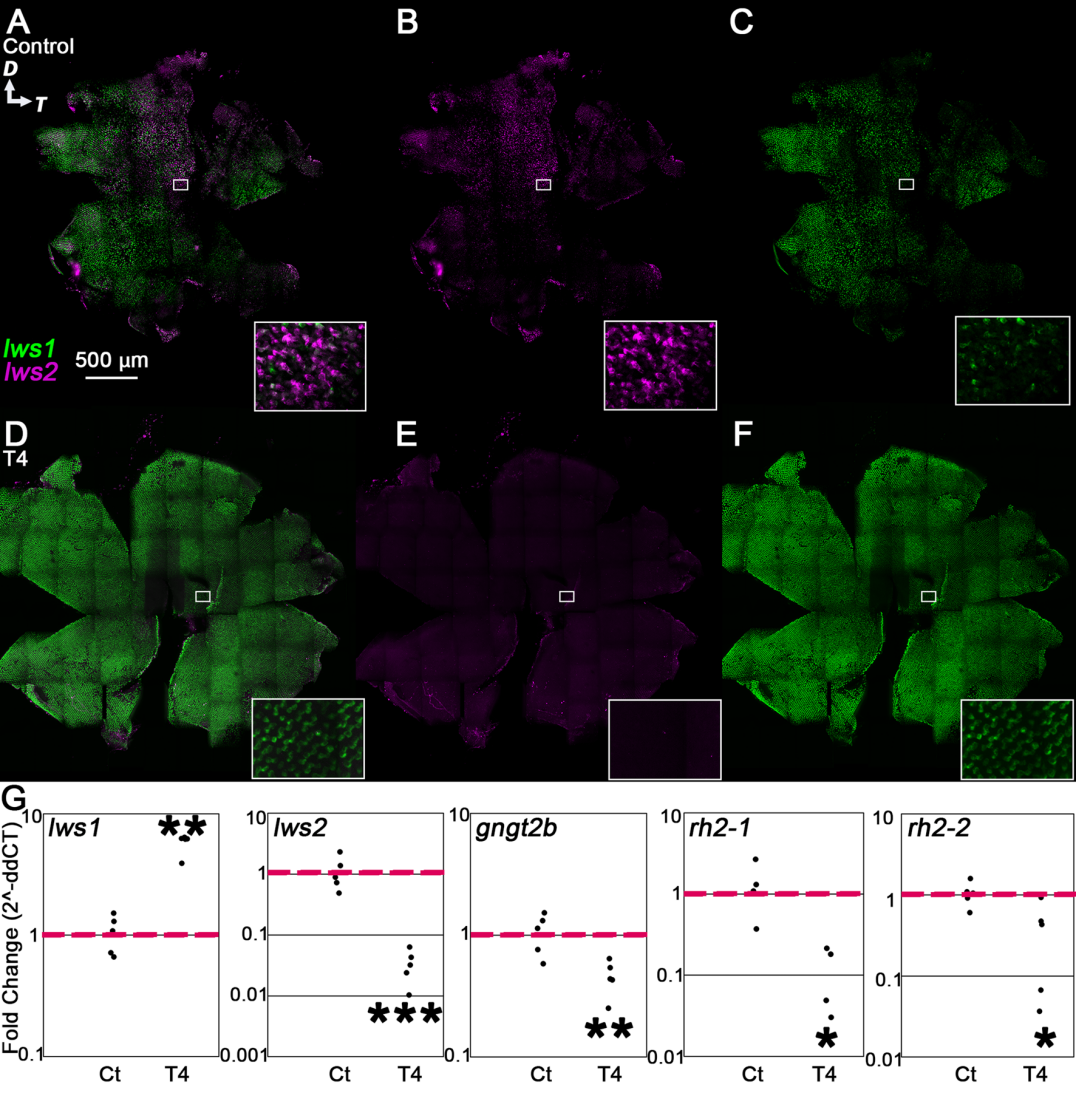
Plasticity of cone transcripts in response to 5 days of T4 treatment. (**A**–**F**) Projections of representative whole, imaged retinas from fish treated with NaOH (control, **A**–**C**) or T4 (treated, **D**–**F**) followed by HCR in situ. Insets show indicated regions enlarged by 1400%. (**A**,**D**) All imaging channels merged. (**B**,**E**) *lws2* (**C**,**F**) *lws1*. Note expanded *lws1* domain and lack of *lws2* mRNA in T4-treated retina. (**G**) qPCR of whole adult eyes. (*lws1*) n = 5, *p* = 0.0079 (Mann–Whitney). (*lws2*) n = 5, *p* = 1.2749E−05 (t-test). (*gngt2b*) n = 5, *p* = 0.0025 (t-test). (*rh2-1*) n = 4, *p* = 0.0168 (t-test). *rh2-2*) n = 5, *p* = 0.0355 (t-test). *D* dorsal, *T* temporal.

Confocal imaging of retinas that underwent hybridization chain reaction (HCR) in situ for *lws1* and *lws2* revealed that after 5 days of T4 treatment, the *lws1* expression domain expanded to a similar extent seen in the *lws:PAC(H)* reporter transgenic (Fig. 2A–F; Supplementary Fig. S2). Interestingly, *lws2* transcript was undetectable by HCR after 5 days of T4 treatment (Fig. 2E). Because our previous study identified the increase in *lws1-*expressing cones as the result of individual cones switching opsins^14^, and because our current results obtained from the *lws:PAC(H)* transgenics showed widespread coexpression of GFP reporting *lws1* and RFP reporting *lws2*, we interpret that the expanded *lws1* domain and lack of *lws2* transcript was likely due to opsin switching. In other words, cone photoreceptors that previously expressed *lws2* have switched to express solely *lws1*. These results also suggest that the GFP:RFP coexpression seen in the *lws:PAC(H)* fish (Fig. 1D) was likely due to slow degradation of the dsRedExpress reporter. Further, qPCR data (Fig. 2G) showed that after 5 days of T4 treatment, *lws1* transcript abundance increased, while *lws2* transcript abundance significantly decreased, in agreement with the HCR confocal images.

We next analyzed other genes known to exhibit altered transcriptional abundance or expression domains after TH treatment; namely *gngt2b*, *gngt2a*^20^, *rh2-1*, and *rh2-2*^14^. We found that the abundance of *gngt2b*, encoding a gamma subunit of transducin associated with LWS2 cones and other cone subtypes in the central retina^26,41^, and known to be downregulated by T3 treatment in larvae, decreased after 5 days of T4 treatment of adults (Fig. 2G)^20^. This finding suggests that cone photoreceptor genes other than those encoding opsins remain plastic to the effects of TH even in adulthood. We also analyzed *gngt2a*, which encodes a paralogous gamma subunit associated with LWS1 cones^20,41^, and which showed an altered expression domain following T3 treatment in larvae, although transcript abundance did not change^20^. In T4 treated adults, abundance of this transcript was not altered (Supplementary Fig. S3). The *rh2* array is another set of tandemly replicated opsins in zebrafish^13^, and the members of this array are known to be affected by TH treatment in larval and juvenile fish^14,20^. We found that exogenous T4 treatment of the adults resulted in decreased abundance of both *rh2-1* and *rh2-2* transcripts (Fig. 2G).

### T4 treatment induces widespread cone transcriptional changes in as little as 24 h

**Figure 3:**
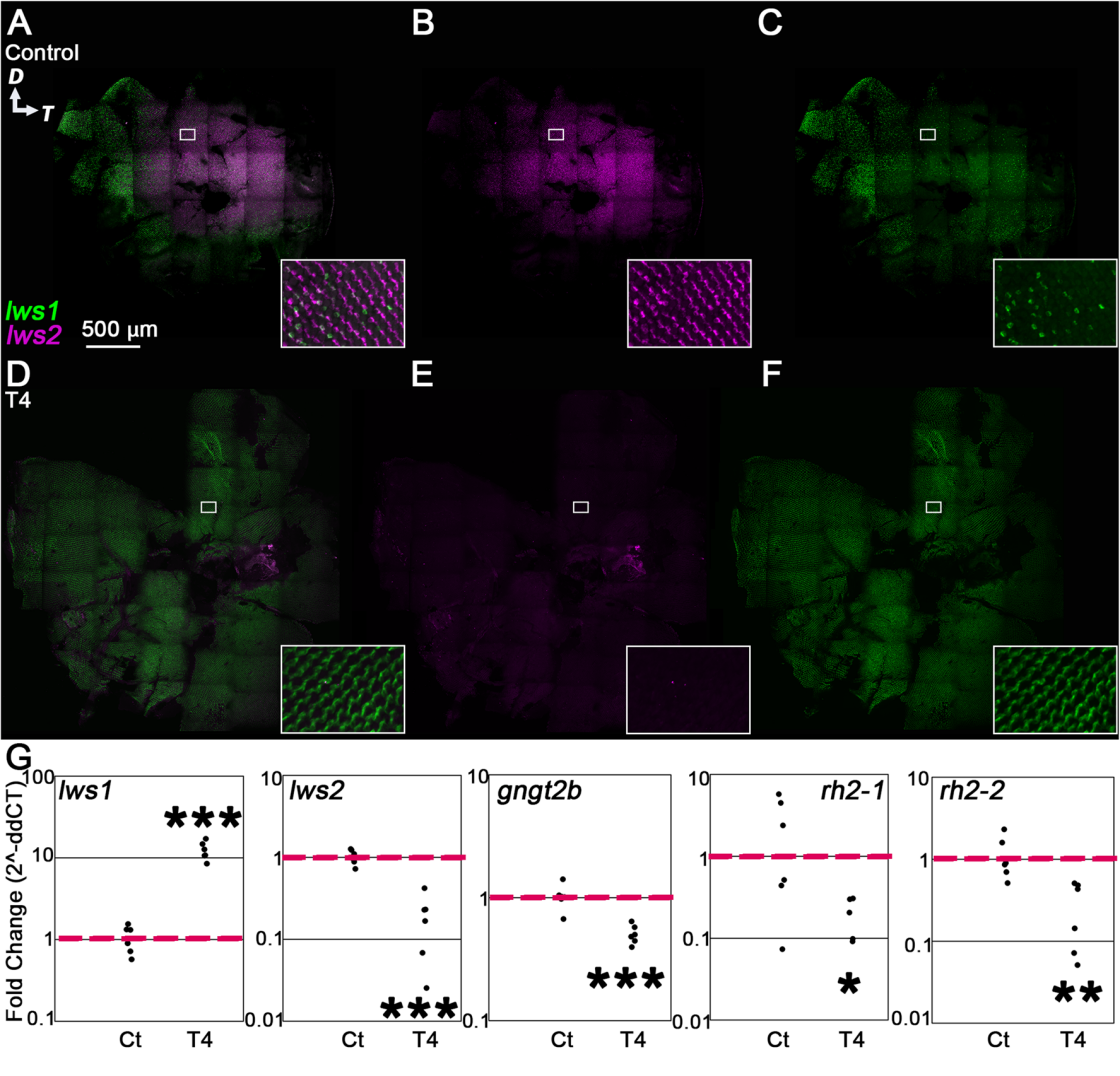
Plasticity of cone transcripts in response to 24-h T4 treatment. (**A**–**F**) Projections of representative whole, imaged retinas from fish treated with NaOH (control, **A**–**C**) or T4 (treated, **D**–**F**) followed by HCR in situ. Insets show indicated regions enlarged by 1400%. (**A**,**D**) All imaging channels merged. (**B**,**E**) *lws2* (**C**,**F**) *lws1*. Note expanded *lws1* domain and lack of *lws2* mRNA in T4-treated retina. (**G**) qPCR of whole adult eyes, n = 6 for each group. (*lws1*) *p* = 1.3235E−07 (t-test). *lws2*) *p* = 0.0008 (t-test). (*gngt2b*) *p* = 0.0002 (t-test). (*rh2-1*) *p* = 0.0233 (t-test). (*rh2-2*) *p* = 0.0065 (t-test). *D* dorsal, *T* temporal.

We then tested whether shorter treatments would also generate changes in transcription of cone genes. Interestingly, the results of the 24-h treatment were similar to those of the 5-day experiment, suggesting that gene expression in cone photoreceptors responds rapidly to changes in T4 levels (Fig. 3A–F; Supplementary Fig. S2). We found that after 24 h of T4 treatment, the *lws1* expression domain expanded to cover the entire retina and *lws2* expression became undetectable by HCR (Fig. 3D–F). The 24-h qPCR results were also similar to the 5-day results, showing that the transcript abundance of *lws1* increased after treatment while the abundance of *lws2, gngt2b, rh2-1 and rh2-2* transcripts significantly decreased (Fig. 3G), and that of *gngt2a* did not change (Supplementary Fig. S3).

### Twelve hours or less of T4 treatment alters lws1 expression, but not the expression of other T4-regulated transcripts

**Figure 4:**
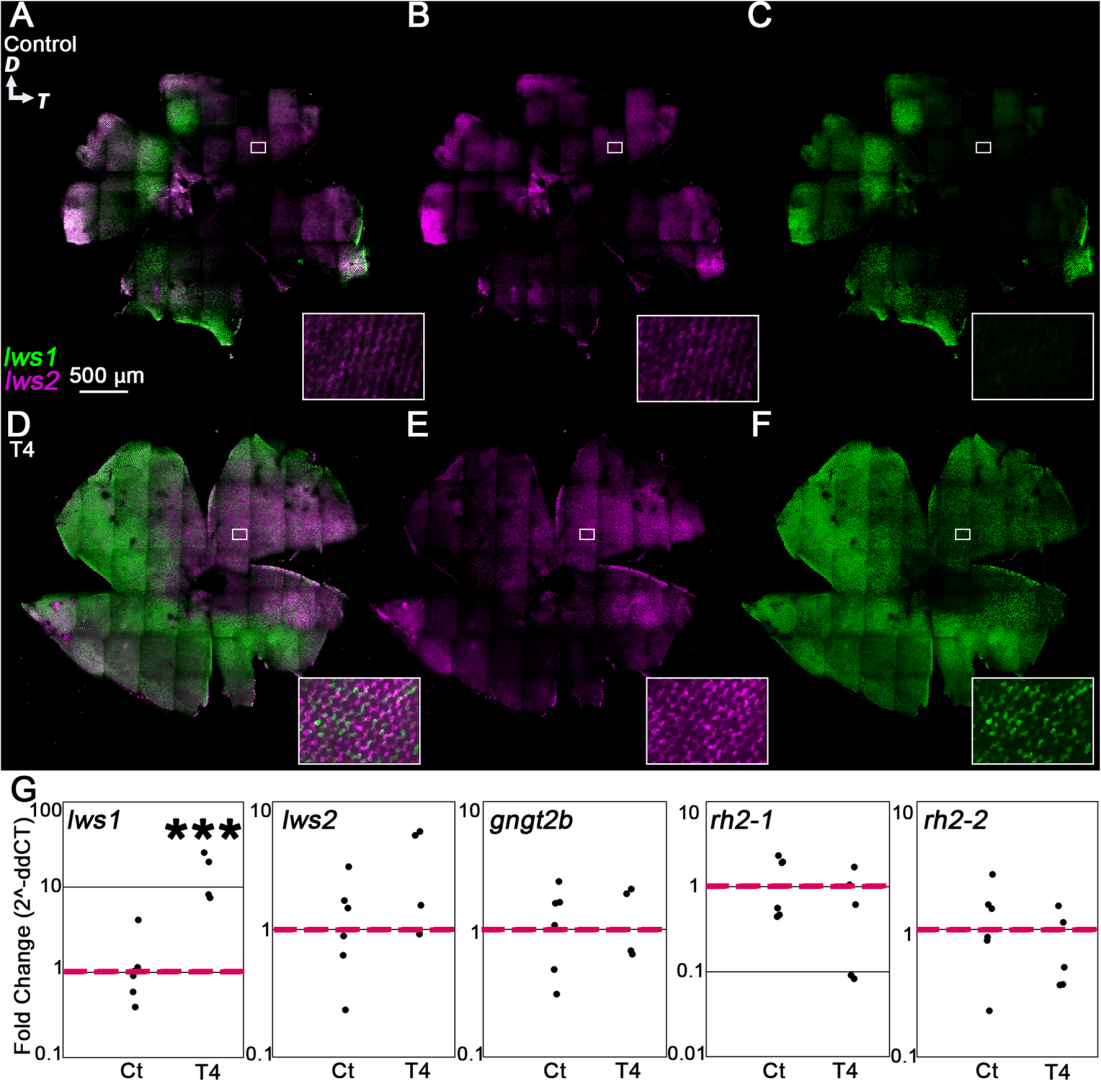
Plasticity of cone transcripts in response to 12-h T4 treatment. (**A**–**F**) Projections of representative whole, imaged retinas from fish treated with NaOH (control, **A**–**C**) or T4 (treated, **D**–**F**) followed by HCR in situ. Insets show indicated regions enlarged by 1400%. (**A**,**D**) All imaging channels merged. (**B**,**E**) *lws2* (**C**,**F**) *lws1*. Note expanded *lws1* domain and colocalization of *lws1* and *lws2* mRNA in T4-treated retina. (**G**) qPCR of whole adult eyes. (*lws1*) n = 6 (control), 4 (treated); *p* = 0.0006 (t-test). (*lws2*) n = 6 (control), 4 (treated); *p* = 0.1424 (t-test). (*gngt2b*) n = 6 (control), 4 (treated); *p* = 0.7863 (t-test). (*rh2-1*) n = 6 (control), n = 5 (treated); *p* = 0.1860 (t-test). (*rh2-2*) n = 6 (control), n = 5 (treated); *p* = 0.3820 (t-test). *D* dorsal, *T* temporal.

Confocal imaging of retinas that underwent HCR in situ revealed that after 12 h of T4 treatment, the *lws1* expression domain expanded to a similar extent seen in 5 day and 24-h treatment groups (Fig. 4A–F; Supplementary Fig. S2). In contrast, however, the *lws2* expression domain remained similar to that of controls (Fig. 4B,E; Supplementary Fig. S2). We found that most of the cells in which *lws2* mRNA was detected also showed *lws1* expression (Fig. 4D). Because mRNA half-lives in vertebrates exhibit a wide range, from minutes to several hours, these results could indicate that many LWS cones actively transcribe both opsin genes after 12 h of T4 treatment, or that the cones have switched to express *lws1* while *lws2* mRNAs remain^42,43^. The specific half-lives of cone opsin mRNAs have not yet been determined, to our knowledge. Our qPCR results corroborate our in situ data, showing a significant increase in *lws1* transcript abundance but no change in *lws2* transcript abundance (Fig. 4G). Further, the transcript abundance of the other genes we investigated (*gngt2b, gngt2a, rh2-1, rh2-2*) also did not change (Fig. 4G; Supplementary Fig. S3). These results suggest that 12 h is not sufficient for all T4-mediated transcriptional changes to be made in adult retina.

**Figure 5:**
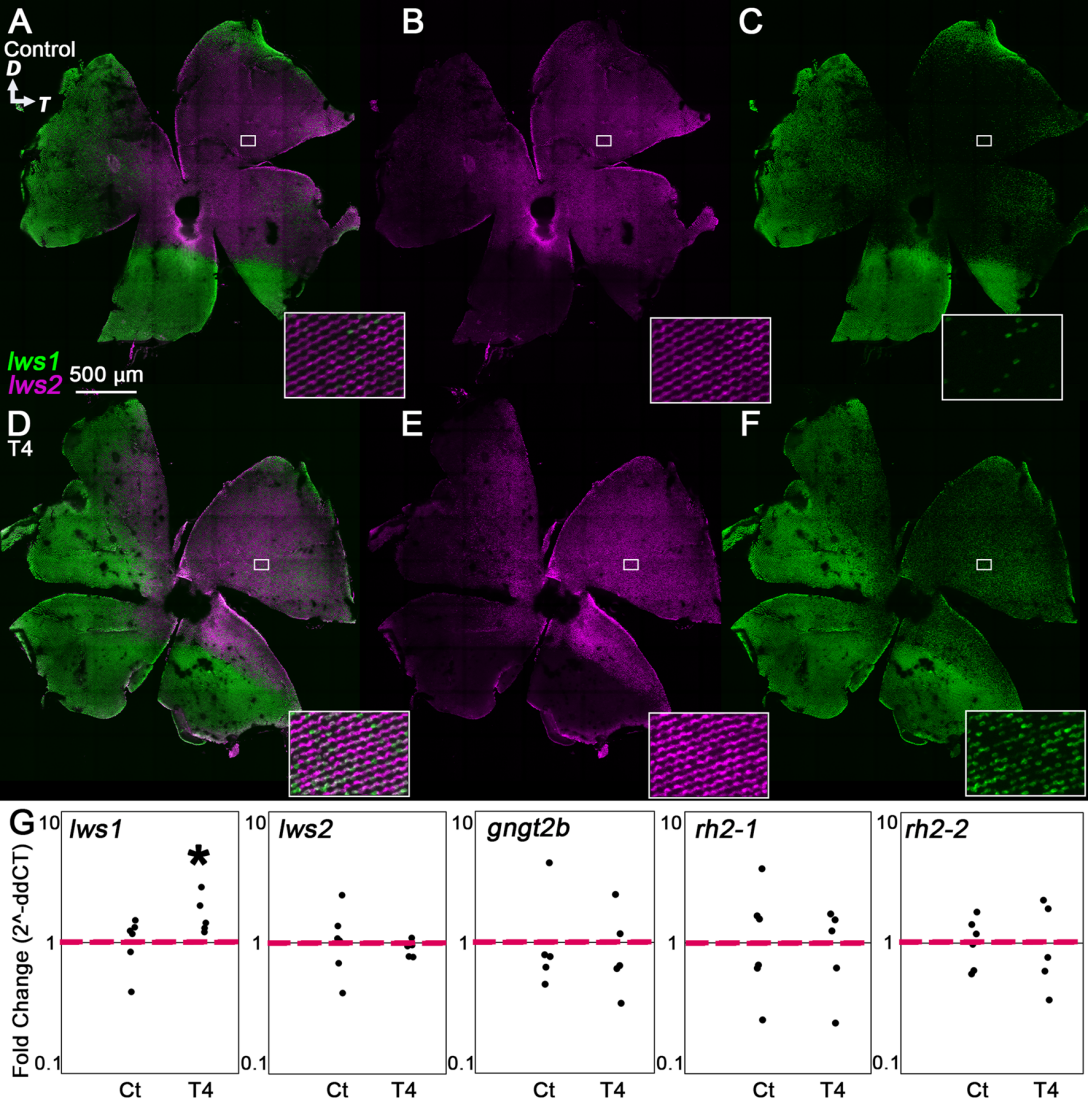
Plasticity of cone transcripts in response to 7-h T4 treatment. (**A**–**F**) Projections of representative whole, imaged retinas from fish treated with NaOH (control, **A**–**C**) or T4 (treated, **D**–**F**) followed by HCR in situ. Insets show indicated regions enlarged by 1400%. (**A**,**D**) All imaging channels merged. (**B**,**E**) *lws2* (**C**,**F**) *lws1*. Note expanded *lws1* domain and colocalization of *lws1* and *lws2* mRNA in T4-treated retina. (**G**) qPCR of whole adult eyes. (*lws1*) n = 6 (control), 5 (treated); *p* = 0.0373 (t-test). (*lws2*) n = 6 (control), 5 (treated); *p* = 0.3173 (Mann–Whitney). (*gngt2b*) n = 5 (both treatments); *p* = 0.8277 (t-test). (*rh2-1*) n = 6 (control), n = 5 (treated); *p* = 0.8246 (t-test). (*rh2-2*) n = 6 (control), n = 5 (treated); *p* = 0.8941 (t-test). *D* dorsal, *T* temporal.

Confocal imaging of adult retinas that underwent HCR in situ revealed that after 7 h of T4 treatment, the *lws1* expression domain expanded to a similar extent seen in the previous treatment groups and the *lws2* expression domain remained similar to that of controls (Fig. 5A–F; Supplementary Fig. S2). We found that most of the cones in which *lws2* mRNA was detected also showed *lws1* expression (Fig. 5D). Our qPCR results corroborate these data, showing a significant increase in *lws1* transcript abundance but no change in *lws2* transcript abundance (Fig. 5G). Further, the transcript abundance of the other genes we investigated by qPCR (*gngt2b, gngt2a, rh2-1, rh2-2*) also did not change (Fig. 5G; Supplementary Fig. S3). These results suggest that 7 h is not sufficient to generate all T4 mediated changes in adult cone transcription.

### Exogenous T4 treatment of adult zebrafish alters skin pigmentation

The adult zebrafish exhibits a characteristic pattern of dark stripes consisting of melanophores (darkly pigmented cells) and iridophores (iridescent cells) alternating with light interstripes containing xanthophores (yellow/orange pigmented cells) and iridophores^44^. Previous work by others has shown that TH is instrumental in determining the pigmentation patterns present in zebrafish skin and maintaining proper pigmentation patterns in juveniles and adults^33,38^. Ablating the thyroid of larval zebrafish resulted in significant skin pigmentation changes after 6 months, particularly an increased number of melanophores and wider stripes^33^. Additionally, a genetically hyperthyroid mutant (*opallus*) showed an increased number of xanthophores and decreased number of melanophores^33^. The *opallus* mutant experiences hyperthyroidy from a very early age. Therefore, we saw the opportunity to test whether the adult pigmentation patterns of euthyroid zebrafish were plastic to more “acute” hyperthyroidy through treatment of adults with T4.

**Figure 6:**
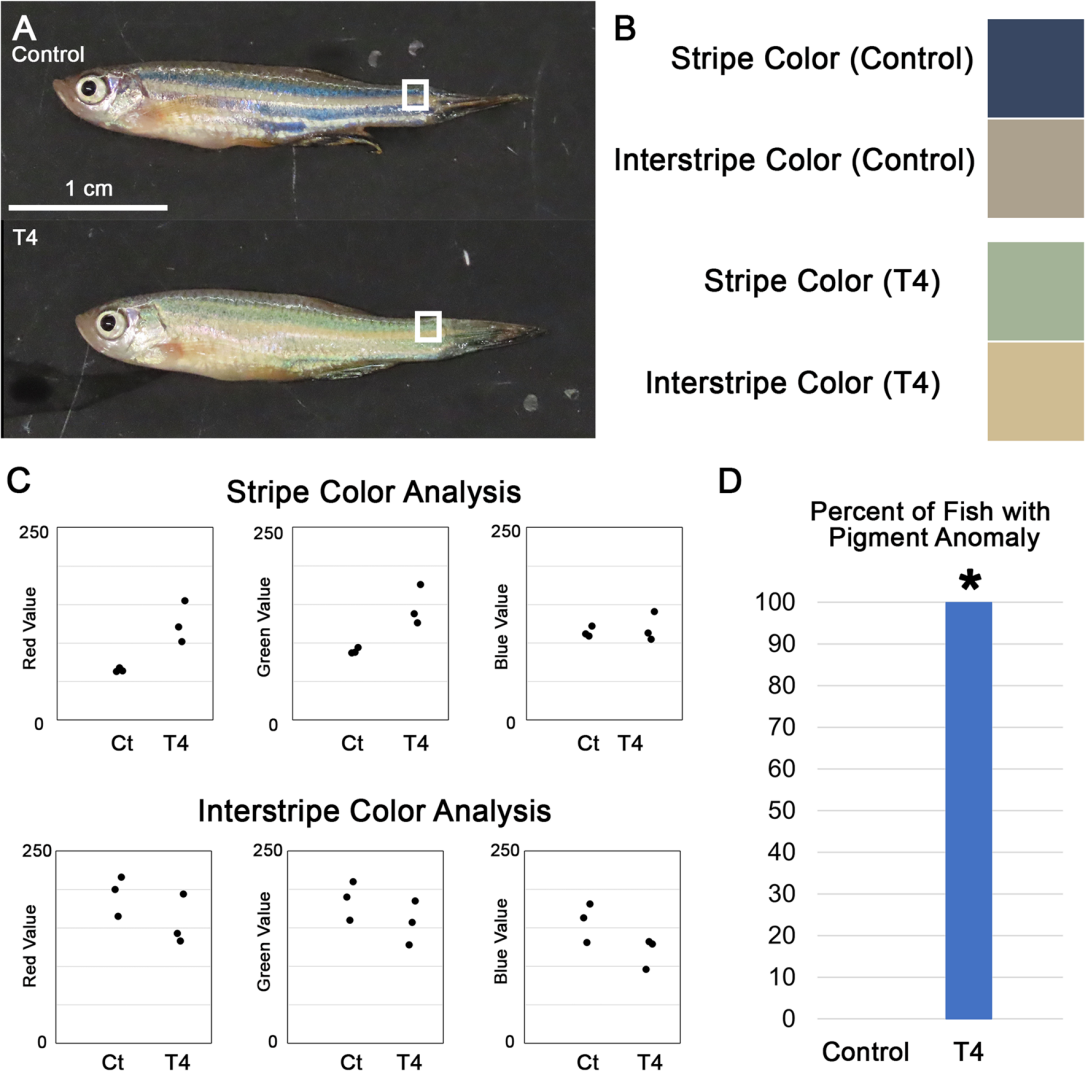
Five day T4 treatment of adult zebrafish alters skin pigmentation. (**A**) Brightfield photographs of representative fish. (**B**) Examples of representative stripe and interstripe colors of control and treated fish. Colors were sampled from regions denoted by rectangles in (**A**). (**C**) Color analysis of stripe and interstripe Red, Green, and Blue scale values, n = 3. Values denote “redness,” “greenness,” and “blueness” on a scale from 0 to 255. High red values indicate high redness. Low red values indicate green, low green values indicate red, low blue values indicate orange. Note trend of higher red and green values in stripes of T4-treated fish. (**D**) Percentages of fish in control and treated groups with altered pigmentation compared to untreated wildtype. All T4-treated fish showed altered skin pigmentation while all control fish showed normal pigmentation, *p* = 0.0143 (proportion test).

We found that WT zebrafish exhibited striking skin pigmentation changes after 5 days of TH treatment. We observed that in T4-treated fish, the dark stripes appeared to lighten and appear green (Fig. 6A,B), and the fish pigment patterns appeared qualitatively similar to the *opallus* mutant^33^. Using photoshop assisted spectroscopy^45^, there appeared to be trends toward differences in stripe color but not interstripe color (Fig. 6C). This change did not occur in the control group (Fig. 6D). These results suggest that skin pigmentation in zebrafish is plastic to external signals even at the adult stage and in response to only 5 days of exposure, and that specific TH levels are required to maintain homeostasis in skin pigmentation.

## Discussion

Cone subtype patterning in the adult zebrafish is plastic. Our results show that exogenous TH treatment alters opsin expression in as little as 7 h. When adult fish were treated with T4 for 24 h or longer, the expression of several genes was affected. *Lws1* expression increased while the expression of *lws2, gngt2b, rh2-1,* and *rh2-2* decreased. Further, we found that when adult fish were treated with T4 for 12 h or less, *lws1* expression increased but the expression of the other transcripts tested did not change. Additionally, we found that exogenous T4 treatment for 5 days alters skin pigmentation in adult zebrafish. Because the appearance of the T4-treated fish was similar to that of the adult *opallus* mutants, and the *opallus* mutants exhibit increased xanthophore numbers and decreased melanophore numbers^33^, it is likely that the T4 treatment also increased xanthophore numbers and decreased melanophore numbers. Our results provide further evidence that homeostatic TH levels are required for zebrafish to maintain normal melanophore and xanthophore populations, and to maintain the opsin expression patterns characteristic of adult zebrafish.

We focused this study upon selected cone photoreceptor transcripts (*lws1, lws2, gngt2a, gngt2b, rh2-1, and rh2-2*), and found that these remain plastic to TH treatment in the adult zebrafish. However, the plasticity of other transcripts such as *rh2-3* and *rh2-4* or *sws1* and *sws2* remains unknown. As T3 levels in a particular cell can be specifically tuned by local deiodinase enzymes, it is possible that cells in various regions of the retina effectively received different amounts of TH^27^. Additionally, data for our 7 h experiment were collected at a different time of day than our other experiments (Supplementary Fig. S4). As zebrafish opsin expression exhibits circadian changes, with opsins expressed at low levels in the morning and high levels in the evening^46–49^, this represents a confounding variable in our evaluation of the 7 h treatment results. In pigmentation analysis, our methods were limited to photography and Photoshop-assisted spectroscopy. Analyses using pigment cell counts or spectrometry would strengthen these results.

The present study advances the extensive literature examining the role of TH in altering or maintaining cone phenotypes in fish. TH signaling has been shown to be important prior to, or during smoltification, a post-embryonic life stage transition that occurs in salmonids as juvenile fish change habitat^50,51^. This transition is accompanied by changes in skin pigmentation (lightening) and cone photoreceptor subtype patterning (long wavelength-shifting)^52–54^. In both coho salmon and rainbow trout, TH signaling is associated with a UV to blue shift in opsin expression that begins toward the end of yolk sac absorption when there is a surge in TH^22,53^. TH signaling also underlies changes in opsin expression during metamorphoses of two flounder species^55,56^. Further, TH has been shown to induce the expression of *cyp27c1*, an enzyme within the retinal pigmented epithelium that converts vitamin A1 to vitamin A2, and thereby long wavelength-shifting pigment sensitivity^14,25,57^. In coho salmon, a chromophore shift naturally occurs and is associated with seasonal variables^58^. Interestingly, there is some evidence that TH-induced plasticity in photoreceptor gene expression may be conserved in humans. Cakir et al.^59^ found that adult patients treated for hypothyroidism showed significant improvement in their color contrast sensitivity, indicating that cone gene expression in the adult human retina may also be plastic to TH levels. The ability of TH to alter gene expression in the photoreceptors of an adult organism could inform therapeutic approaches to disorders involving cone photoreceptors, or in determining optimal protocols for retinal organoid development or other photoreceptor replacement strategies.

Thyroid hormone receptors (*thraa*, *thrab*, *thrb*), deiodinases (*dio1*, *dio2*, *dio3a*, *dio3b*) and thyroid hormones (T3/T4) are present in the adult zebrafish retina^39,57^; however, to our knowledge the topographical distributions of these remain unknown. Retinoic acid signaling, however, is known to occur in the ventral portion of the retina in embryonic, larval^60–62^, and juvenile (1 month) zebrafish^36^. In thyroid-ablated juvenile *lws:PAC(H)* reporter fish, *lws1*:GFP+ cones are only present in a ventral patch that correlates with the location of RA signaling, showing that in the absence of TH, RA signaling is sufficient to promote the expression of *lws1*^14^. Further studies are needed to examine the topography of both TH and RA signaling and their signaling components in the zebrafish, particularly in adult retinas.

Based on results from our previous studies in larval zebrafish, our data suggest that the cones co-expressing *lws1* and *lws2* identified in our 12 and 7 h experiments are likely undergoing opsin switching^14^. The results from the present study additionally reveal that the kinetics of T4 treatment and changes in gene expression within adult retinas are complex. Since zebrafish have two loci for each specific opsin gene (all are on autosomes), the coexpression could be achieved either by the expression of both genes on each locus, or by the expression of one gene from one locus and the other gene from the second locus. Alternatively, it is possible that *lws2* was not being transcribed and the mRNA from hours before had not yet been degraded. Other factors, including a temporal difference in the mechanisms controlling *lws1* versus *lws2* expression could also underly this observation. For example, the mechanism for regulating *lws1* may involve direct interaction of a liganded TH receptor with elements on the *lws1* locus, while control of *lws2* may be indirectly mediated and/or require recruitment of additional co-repressors. Interestingly, in larvae, 24 h of treatment with 100 nM T3 produced similar results to the adult 12-h treatment, in which *lws1* levels increased while *lws2* levels did not change, but by 48 h of larval treatment, *lws2* levels decreased^14^. The faster rate of TH-induced changes in adults could be due to the higher concentration of TH in the system water (100 nM for larvae vs. 386 nM for adults), or other factors such as changes in the temporal controls of TH response between larvae and adults.

While the exact nuclear hormone receptors responsible for controlling *lws1* vs *lws2* gene expression remain unknown, TH receptor beta 2 (*thrb2*) is a reasonable candidate for this role. *Thrb2* is required for LWS cone development in zebrafish^57,63,64^ and sequences having predicted TH receptor binding activity have been identified near the *lws1* and *lws2* genes^14^. Additionally, overexpression of *thrb2* in zebrafish cones and bipolar cells has been shown to long wavelength-shift the sensitivity of the adult retina, with the response data fitting a vitamin A1-based model with maximal amplitudes near the measured amplitudes for zebrafish LWS1 cones^13,65^. Adult zebrafish retinas experience several surges of TH during the zebrafish lifespan^33,57,66^, and so the shift from LWS2 cones to LWS1 cones in the *thrb2* overexpression model may represent the consequences of predominantly liganded TH receptor. The complex kinetics of TH-mediated changes in adult photoreceptor gene expression could be mediated by the ability of the Thrb2 receptor (or other nuclear hormone receptor) to function in a manner that differs from the canonical model of nuclear hormone receptor action, in which the presence of ligand leads to recruitment of coactivators and the absence of ligand leads to recruitment of corepressors at genes that are positively regulated by TH^31^. Indeed, it has been shown that both liganded and unliganded forms of Thrb2 can promote transcription of positively regulated genes^67^, although the activity level of liganded receptor is higher. Further, *thrb1*, a splice variant of *thrb2,* has been shown to control gene expression by altering ratios of coactivators and corepressors, rather than recruiting either coactivators or corepressors^68^. Roles for TH receptors other than Thrb2 are also possible; for example, TRα has been implicated in UV-to-blue-sensitive opsin switching in salmonids^21^, and during TH-induced metamorphic changes in opsin expression in the winter flounder^56^. The results shown here emphasize the necessity for the identification and study of the transcription factors that regulate tandemly replicated opsin genes.

We observed that both *rh2-1* and *rh2-2* were downregulated by TH treatment in adults. In contrast, *rh2-2* is upregulated by exogenous TH in larvae and unchanged in juveniles^14^. While the expression domains of the *lws* and *rh2* opsins shift through the juvenile stage, the expression domain of *rh2-2* is particularly dynamic as the fish grows/ages^34^. In the embryo, *rh2-2* is expressed both centrally and peripherally. In the juvenile, *rh2-2* is expressed in the dorsal periphery and ventral mid-periphery, and in adults, *rh2-2* is expressed centrally^34^. Previous work has shown how cis elements of the *rh2* array underly expression domains of the *rh2* genes in adult zebrafish^69^. Our results here provide additional insight into how TH may also be involved in tuning the expression of *rh2* genes, and that this tuning effect may change over the zebrafish lifespan.

In addition to regulating cone opsin gene expression and the type of opsin chromophore, TH has been shown to regulate multiple photoreceptor transcripts, including multiple transcripts that are differentially expressed in LWS1 and LWS2 cones such as *gngt2b*^20^. Recent work has shown that zebrafish cone subpopulations exhibit inter-population transcriptional heterogeneity and intra-population heterogeneity, with *gngt2b* as an example of a transcript that varies in expression between the LWS cone subtypes, and within the cone subtypes^20,26^. Other work has implicated *thrb2* in regulating multiple genes that are expressed in spatial gradients in mouse retina^70^. Taken together, these results implicate TH as an important regulator of transcriptional heterogeneity in cone populations. Because the non-opsin, LWS2 cone-enriched gene *gngt2b* exhibited plasticity to TH treatment in adult fish, it is possible that additional cone-expressed genes also remain sensitive to TH treatment in the adult. Therefore, TH may be involved in regulating transcriptional heterogeneity among and between cone subpopulations in adult zebrafish.

TH signaling is an important regulator of life history transitions in fish^50^ and other vertebrates such as frogs^71^. Many of these life stage transitions are accompanied by a change in habitat or ecological niche, requiring different visual system capabilities^52,55,56,71^. Zebrafish also undergo TH-mediated changes in jaw morphology, pigmentation, and feeding strategy as they change from larvae to juveniles^32,72,73^. The plasticity we observed in adults, however, cannot be directly explained as relevant to a life history change, as we used captive, adult, reproductively mature zebrafish. This plasticity could instead indicate that TH signaling serves as an ongoing mechanism for maintaining cone subtype patterning in adults. Indeed, TH gradients in adult mice are involved in maintaining cone subtype patterning^15,24^. As zebrafish possess the ability to regenerate their retinas after injury, the plasticity of adult cones could also be important in re-establishing some elements of cone subtype patterning in the regenerating retina^74,75^. There is evidence that zebrafish re-establish near-normal topographic patterns of *lws1* vs *lws2* after extensive damage to retinal neurons and subsequent retinal regeneration^75^, and TH signaling may underlie this phenomenon. Plasticity within the visual system of cichlids experimentally exposed to different lighting conditions has been demonstrated, although the underlying mechanism(s) are not known^76^. It is possible that non-captive zebrafish utilize an endocrine mechanism to adjust their visual system to changing environmental conditions. For example, cichlids respond to increases in turbidity by long wavelength-shifting visual function^77^, and larval and juvenile Atlantic halibut respond to white-to-blue light environmental change by increasing density of LWS cones. Further, the overall cone photoreceptor pattern transitions from a hexagonal lattice to a square mosaic, likely through opsin switching^78^.

The present study builds upon our previous work showing that TH regulates the expression of the *lws* and *rh2* opsins in larval and juvenile zebrafish, by determining the extent to which the zebrafish retina is plastic to TH, and reveals an interesting difference in the TH response kinetics of *lws1* and other cone photoreceptor genes. We found that skin pigmentation in adult zebrafish also remains plastic to exogenous TH treatment, showing an overall plasticity to TH that is reminiscent of TH-mediated postlarval photoreceptor and pigmentation changes seen in salmonids. This work adds to the body of literature showing TH is an important regulator of retinal development and cone subtype patterning, as well as an essential driver of retina and pigment phenotype changes in fish.

## Methods

### Ethics statement

All animal experiments were performed in accordance with relevant guidelines and regulations and with approval from the University of Idaho Institutional Animal Care and Use Committee. Methods are also reported in accordance with ARRIVE guidelines.

### Animals

Zebrafish were propagated and maintained according to Westerfield, on recirculating, monitored, and filtered system water, on a 14:10 light/dark cycle, at 28.5 °C^35^. Procedures involving animals were approved by the Animal Care and Use Committee of the University of Idaho. Wild-type (WT) zebrafish were of a strain originally provided by Scientific Hatcheries. The *lws:PAC(H)* transgenic line [*Tg(LWS1/GFP-LWS2/RFP-PAC(H))#430,* (kj15Tg)] harbors a PAC clone that encompasses the *lws* locus, modified such that a GFP-polyA sequence, inserted after the *lws1* promoter, reports expression of *lws1*, and an RFP (dsRedExpress)-polyA sequence, inserted after the *lws2* promoter, reports expression of *lws2*^40^. This line was the kind gift of Shoji Kawamura and the RIKEN international resource facility. Adult (0.5–1.5 years) zebrafish were used. Both sexes were represented in each control and treatment group for all experimental endpoints. Zebrafish were considered adult if they were over 6 months of age, showed adult pigmentation patterns, and displayed adult male/female body shape characteristics (slim for males, plump for females)^35^ .

### Thyroid Hormone Treatments

Stock solutions of tetra-iodothyronine (T4; Sigma) were prepared in NaOH (Sigma), and maintained at − 20 °C in the dark. During treatments, adult zebrafish were maintained individually in 250 mL beakers in system water. 10,000× T4 stock solution was added to system water for a final concentration of 386 nM as in Suliman et al.^14,38^ (NaOH final concentration was 0.01% and did not alter system water pH). Controls were treated with 0.01% NaOH. For experiments lasting > 1 day, fish were fed once daily and treatment solution was completely replaced after feeding. Duration of treatments was 7 h, 12 h, 24 h, or 5 days (Supplementary Fig. S4)^14^. T4 (rather than T3 or a synthetic analog) was used as the experimental treatment to be consistent with other studies of TH treatment in postlarval fish^14,25,33,79^, and with the rationale that TH would primarily enter via the gills into the bloodstream, and T4 is the predominant, circulating form of TH^28^. Blinding investigators to treatment condition was not possible due to the need for proper solution changes, overall appearance of the fish for the 5 day treatments (Fig. 6A), and the large increases in *lws1* expression in the T4 treatment groups for all treatment durations (Figs. 1, 2, 3, 4 and 5). Sample sizes are provided in Figure legends; no processed samples were excluded.

### RNA extraction and quantitative RT-PCR (qPCR)

Total RNA from zebrafish eyes was extracted using the Machery-Nagel Nucleospin RNA kit, and then the Superscript III/IV (Invitrogen) was used to synthesize cDNA template with random primers. Gene-specific primer pairs for qPCR are provided in Supplemental Table S1. Amplification was performed on a StepOne Real-Time PCR system using SYBR Green or Power Track SYBR Green master mix (Applied Biosystems). Quantification of transcript abundance was relative to the reference transcript (*β-actin*), using the ddCT method. Transcript abundance for *β-actin* did not vary significantly between control and treated groups. Graphing and statistics were performed in Excel. Sample groups were evaluated for normal distributions using the Shapiro–Wilk test. For comparisons showing normal distributions, *p* values were calculated using Student’s t-test, and for comparisons not showing normal distributions, *p* values were calculated using Mann–Whitney tests. *** denotes *p* < 0.001, ** denotes *p* < 0.01, * denotes *p* < 0.05.

### Histological processing

Fixation and preparation of adult PAC(H) eyes for tissue sectioning were performed as previously described^14,36,74,75^. In brief, zebrafish were humanely euthanized (anesthetized using MS222 overdose followed by decapitation), eyes were removed, corneas pierced and lenses removed. Eyecups were then fixed with 4% paraformaldehyde in a phosphate-buffered, 5% sucrose solution overnight at 4 °C, washed in increasing concentrations of sucrose, cryoprotected overnight at 4 °C in phosphate-buffered 20% sucrose, embedded and frozen in a 2:1 solution of 20% sucrose: OCT medium (Sakura Finetek, Torrance, CA), and sectioned at 5 μm thick^60^ on a Leica CM4050 cryostat.

### Hybridization chain reaction (HCR) in situ hybridization

HCR procedures were carried out according to the manufacturer’s instructions (Molecular Instruments), with the exception that we did not incorporate a proteinase K treatment prior to the post-fixation step. In brief, zebrafish retinas were dissected and fixed overnight in 4% paraformaldehyde in phosphate-buffered saline (PBS) at 4 °C. Tissues were then washed in PBS, dehydrated in MeOH, and stored in MeOH at − 20 °C at least overnight. Tissues were rehydrated in a graded MeOH/PBS/0.1% Tween 20 series, and post-fixed with 4% paraformaldehyde in PBS prior to hybridization. Hybridization was done overnight at 37 °C. Tissues were washed with the manufacturer’s wash buffer, and then 5XSSCT (standard sodium citrate with 0.1% Tween-20), and the amplification/chain reaction steps were performed following the manufacturer’s protocol. Transcript-selective probe sets were designed and generated by Molecular Instruments (Supplementary Table S2) and can be ordered directly from their website.

### Confocal microscopy

Whole, fixed *lws:PAC(H)* adult retinas, cryosections of *lws:PAC(H)* eyecups, and HCR-processed, adult (0.5–1.5 years) WT retinas were mounted in glycerol and imaged with a 20× dry lens using a Nikon–Andor spinning disk confocal microscope and Zyla sCMOS camera running Nikon Elements software (RRID:SCR_014329), and 3 µm-step sizes were used for Z-series images. Z-stacks were flattened by max projection, and brightness/contrast adjusted in FIJI (ImageJ) (RRID:SCR_002285). Multiple images encompassing the entirety of whole retinas or whole cryosections were stitched together using the “large stitched image” feature in Nikon Elements Software.

### Analysis of comparative areas of expression domains

Expression domains were traced using the freehand measurement tool in FIJI/ImageJ as described in Stenkamp et al.^75^. Areas containing predominantly or exclusively GFP+ cones or RFP+ cones were measured within the individual fluorescence channels while areas containing “interspersed GFP+ and RFP+” cones (including areas containing co-labeled cones) were measured using both the red and green channels. Each area was traced in triplicate with the freehand selection tool in FIJI to ensure measurement reproducibility. Percentages were determined by dividing the number of pixels in each expression domain by the number of pixels in the entire retina^75^. This strategy was also applied to measure domains of native *lws1* and *lws2* expression in WT whole retinas imaged after HCR in situs. The Shapiro–Wilk test was used to check for normal distribution. Differences between each measurement were tested using Student’s t-test. Overall difference between control and treatment was tested using Fisher’s Exact Test.

### Brightfield photography and color measurements

Fish were anesthetized using MS-222 and placed on a sterilized portion of the lab bench. Fish were photographed using a Canon PowerShot SX70 HS, and each fish was photographed with and without flash, with a focal length between 40 and 60 mm. Stripe and interstripe colors of individual zebrafish were measured using the RGB spectrum tool in Adobe Photoshop (RRID:SCR_014199)^45^.

### Supplementary Information

Supplementary Information.

## Data Availability

The data presented in this study are available within the paper and the Supplementary materials.
