# Supplementary material for: Plasticity of cone photoreceptors in adult zebrafish revealed by thyroid hormone exposure
Source: Sci Rep. 2023 Sep 21;13:15697. doi: 10.1038/s41598-023-42686-x (PMC10514274; doi:10.1038/s41598-023-42686-x)
Supplement: Supplementary file 1 — Supplementary Information. [file 41598_2023_42686_MOESM1_ESM.pdf]

Supplementary Information for Farre et al., Plasticity of Cone Photoreceptors in Adult Zebrafish Revealed by Thyroid Hormone Exposure

Supplementary Tables

Supplementary Table S1: Primers Used for qPCR

Gene	Forward Primer	Reverse Primer
beta actin	GTACCACCAGACAATACAGT	CTTCTTGGGTATGGAATCTTGC
gngt2a	GTGACCTGTTGCCTCCATCG	TTTAGAGACAGGCTCTCTGGT
gngt2b	ATCCACAGTCAGGATGGCTCG	TCGGCAGATAAACCCTCCAC
lws1	CCCACACTGCATCTCGACAA	AAGGTATTCCCCATCACTCCAA
lws2	AGAGGGAAGAACTGGACTTTCAGA	TTCAGAGGAGTTTTGCCTACATATGT
rh2-1	CAGCCCAGCACAAGAAACTC	AGAGCAACCTGACCTCCAAGT
rh2-2	TTTTTGGCTGGTCCCGATACA	CAGGAACGCAGAAATGACAGC

Supplementary Table S2: Probe Sets Used for HCR

Gene	NCBI Accession Number	Probe Set Size
lws1	NM_001313715.1	13
lws2	NM_001002443.2	9

Supplementary Figures

Supplementary Figure S1

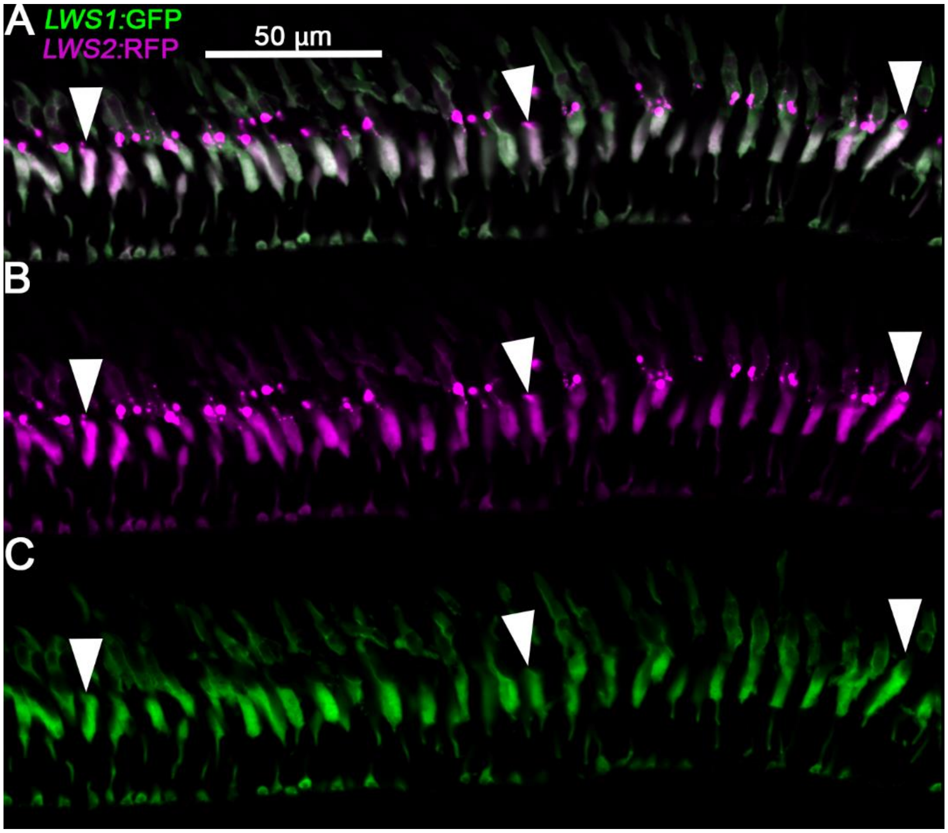

Figure S1: Magnified views of single optical Z-slice (1 µm thick) of individual GFP and RFP imaging channels and their overlay, demonstrating the presence of colabeled cones in a representative section of a T4-treated *lws:PAC(H)* adult zebrafish. A. Merged image. B. RFP only (reporting *lws2*). C. GFP only (reporting *lws1*). Arrowheads are provided to track individual cones through the imaging series. The RFP+ punctae in apical regions of cones likely represent aggregates of dsRedExpress [1].

Supplementary Figure S2

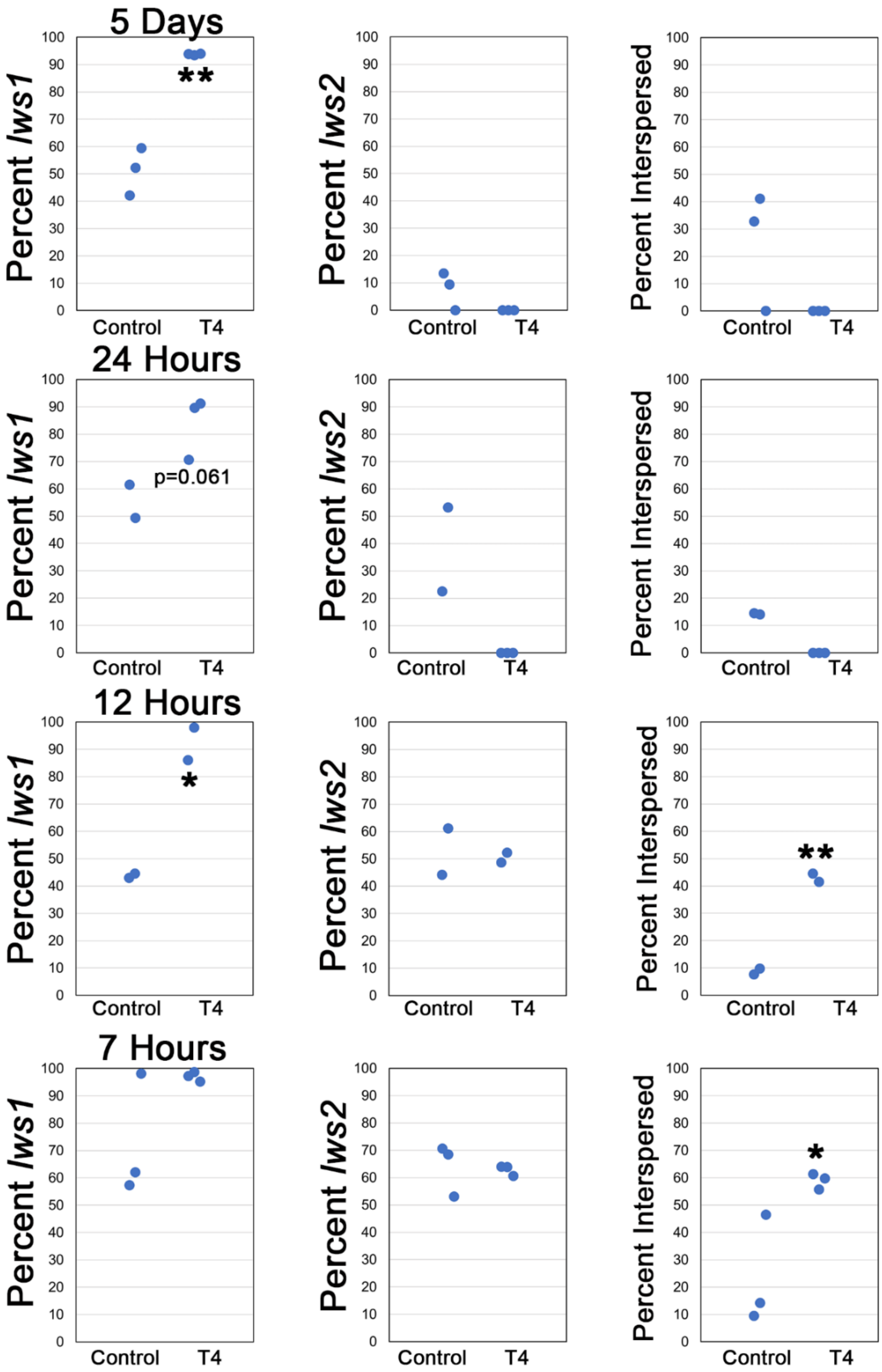

Figure S2: Analysis of areas of expression domains of native *lws1* and *lws2* as detected by HCR in situs. Note that for 5 days and 24 hours, no *lws2* was found by HCR. Sample sizes were too small for

nonparametric tests, so statistics were not run on “percent *lws2*” or “percent interspersed” for 5 days and 24 hours. For the 5 day treatment, *lws1* expression domain expanded from ~51 to ~93% of the retina (p=0.0011). For 24 hours, *lws1* expression expanded from ~55 to ~84% of the retina (p0.0608). For 12 hours, *lws1* expression expanded from ~43 to ~92% of the retina (p=0.0151); *lws2* expression remained the same ~ 50%; and the interspersed and/or coexpressing *lws1*/*lws2* domain increased from ~8 to ~ 43% of the retina. For 7 hours, one control retina showed a remarkably large *lws1* expression domain but was included in statistical analyses. *Lws1* expression showed a trend toward expansion from ~72 to ~97% of the retina (p=0.1301); *lws2* expression remained similar around ~61% of the retina; the interspersed and/or coexpressing *lws1*/*lws2* domain expanded from ~23 to ~59% of the retina (p=0.0392).

<u>Supplementary Figure S3</u>

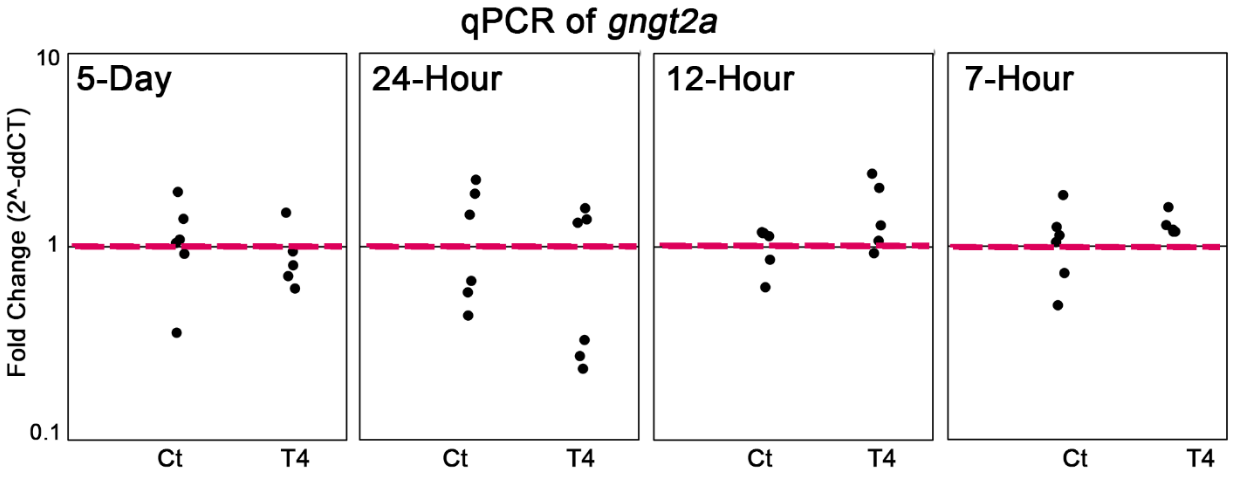

Figure S3: *Gngt2a* expression in control and T4 treated adult zebrafish. qPCR of whole adult eyes. Note that transcript abundance of *gngt2a* (a transcript enriched in LWS1 cones compared to LWS2 cones)[2, 3] did not change in any condition, matching previous results in larvae. Five day) n=6 (control), 5 (treated); p=0.617475 (t-test). 24-hour) n=6 (both treatments); p=0.329755 (t-test). 12-hour) n=6 (control), 5 (treated); p=0.23404 (Mann-Whitney). 7-hour) n=6 (control), 5 (treated); p=0.258549 (t-test).

Supplementary Figure S4

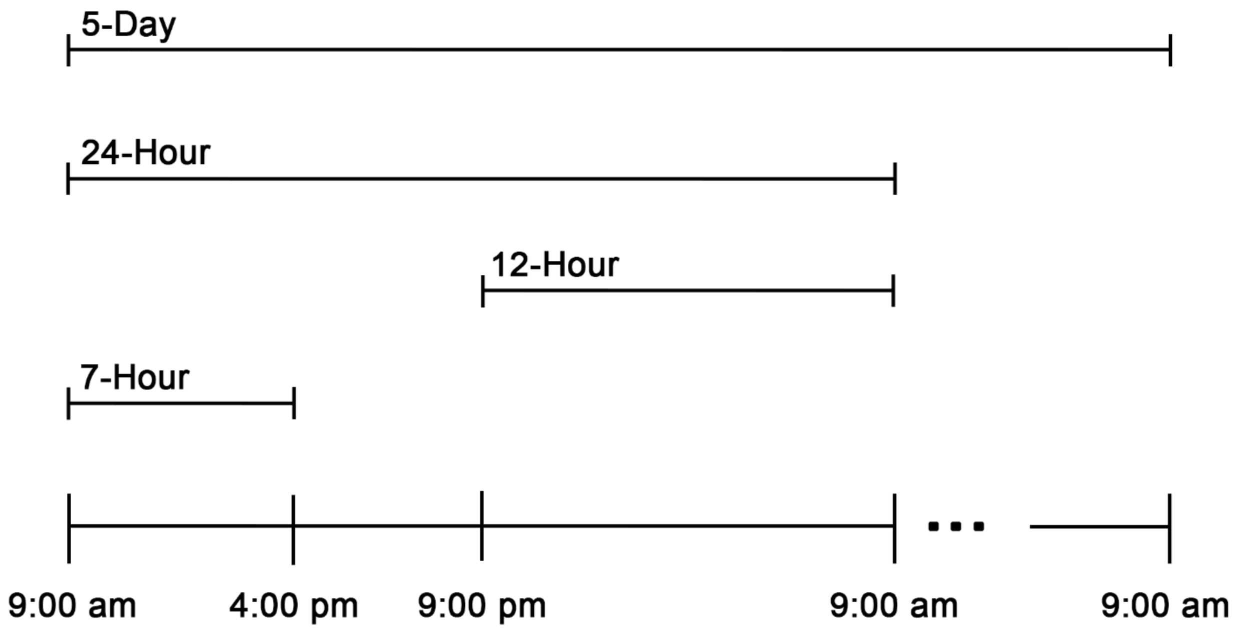

Figure S4: Timeline of adult treatments. Five day and 24-hour treatments began at 9:00 am and ended at 9:00 am. 12-hour treatments began at 9:00 pm and ended at 9:00 am. 7-hour treatments began at 9:00 am and ended at 4:00 pm. Light onset = 8:30 am; Light offset = 10:30 pm.

References

1. Stenkamp, D.L., D.D. Viall, and D.M. Mitchell, *Evidence of regional specializations in regenerated zebrafish retina.* Exp Eye Res, 2021. **212**: p. 108789.
2. Farre, A., et al., *Long wavelength-sensing cones of zebrafish retina exhibit multiple layers of transcriptional heterogeneity.* Front Cell Neurosci, 2023.
3. Ogawa, Y. and J.C. Corbo, *Partitioning of gene expression among zebrafish photoreceptor subtypes.* Sci Rep, 2021. **11**(1): p. 17340.
